# Deletion of AU-Rich Elements within the Bcl2 3′UTR Reduces Protein Expression and B Cell Survival *In Vivo*

**DOI:** 10.1371/journal.pone.0116899

**Published:** 2015-02-13

**Authors:** Manuel D. Díaz-Muñoz, Sarah E. Bell, Martin Turner

**Affiliations:** Lymphocyte Signalling and Development, The Babraham Institute, Cambridge, United Kingdom; Colorado State University, UNITED STATES

## Abstract

Post-transcriptional mRNA regulation by RNA binding proteins (RBPs) associated with AU-rich elements (AREs) present in the 3′ untranslated region (3’UTR) of specific mRNAs modulates transcript stability and translation in eukaryotic cells. Here we have functionally characterised the importance of the AREs present within the Bcl2 3’UTR in order to maintain Bcl2 expression. Gene targeting deletion of 300 nucleotides of the Bcl2 3’UTR rich in AREs diminishes Bcl2 mRNA stability and protein levels in primary B cells, decreasing cell lifespan. Generation of chimeric mice indicates that Bcl2-ARE^∆/∆^ B cells have an intrinsic competitive disadvantage compared to wild type cells. Biochemical assays and predictions using a bioinformatics approach show that several RBPs bind to the Bcl2 AREs, including AUF1 and HuR proteins. Altogether, association of RBPs to Bcl2 AREs contributes to Bcl2 protein expression by stabilizing Bcl2 mRNA and promotes B cell maintenance.

## Introduction

Human B-cell lymphomas are characterised by sequential genetic alterations that deregulate several pathways, including cell cycle and apoptosis. Genetic translocation affecting proto-oncogenes, such as Bcl2, Bcl6 or c-Myc, are found in many tumours, including follicular B-cell lymphoma, Burkitt lymphoma and “double hit” (DH) mature B cell lymphomas [1,2]. In most of the cases, genetic juxtaposition of the oncogene to an enhancer of the Ig locus is the cause of increased gene expression. For example, the Bcl2 translocation t(11,14) is commonly found in follicular-center B lymphomas. This translocation places the Bcl2 gene under the Eμ enhancer of the Igh locus [3]. Enhanced transcription and expression of Bcl2 increases B cell survival [4] and is important for maintenance and progression of tumours [5]. Endogenous expression of Bcl2 is not required for the development of Eμ-myc induced B-cell lymphoma, but it is needed to maintain mature B cells in healthy mice [6,7].

While gene transcription is anomalous in many tumours, post-transcriptional gene regulation may remain intact. Chemical modulation of the different post-transcriptional regulatory mechanisms offers alternative drug-targeting opportunities to reduce oncogene protein expression. RNA molecules are associated with RNA binding proteins (RBPs) during and after their transcription. RBPs control RNA splicing, transport, location, stability and translation, modulating the nature and content of proteins within the cell. Many mRNA encoding proto-oncogenes are subjected to post-transcriptional regulation, including c-Myc, Bcl6 and Bcl2 [8–10]. Within the 3′UTR of these mRNAs, multiple adenine uridine (AU)-rich elements (ARE), including pentamers (AUUUA) and nonamers (UUAUUUAUU), are bound by AU-rich binding proteins (AUBPs), which modulate mRNA stability in a target-dependent manner [11].

Post-transcriptional regulation of Bcl2 mRNA is thought to be a key element for Bcl2 protein expression. The 3′UTR of Bcl2 mRNA is considerably longer than the coding sequence and contains several binding motifs for RBPs and microRNAs. In particular, the binding of AUBPs to the AREs present in the proximal region after the stop codon has been functionally implicated in controlling the fate of Bcl2 mRNA (this 300 bp long sequence is described in the manuscript as the Bcl2 ARE-rich sequence). Different biochemical studies suggest that the binding of different AUBPs to Bcl2 AREs exerts opposing effects on Bcl2 mRNA stability. HuR, nucleolin and ErbB3 (Ebp1) may act as mRNA stabilizers, whereas AUF1, Mex3D (Tino) and Tis11b may promote Bcl2 mRNA degradation *in vitro* [12–16]. Recently, the generation of specific knockout (KO) mice for HuR and AUF1 have shown that both proteins may contribute to Bcl2 mRNA stabilization in B cells [17,18]. Thus, contradictory results have been achieved from *in vitro* and *in vivo* studies, and the importance of post-transcriptional regulation of Bcl2 mRNA for final protein expression remains unclear. In this study, we have evaluated the impact of the genetic deletion of the Bcl2 ARE-rich sequence on Bcl2 expression in primary B cells. We demonstrate that the binding of RBPs to this sequence of the 3’UTR is directly linked to the stabilization of the Bcl2 mRNA and regulates Bcl2 protein expression with functional consequences for B cell maintenance *in vivo*.

## Materials and Methods

### Generation of Bcl2-ARE^flox/flox^ mice

Bcl2-ARE^flox/flox^ (Bcl2^tm1Tnr^) conditional mice were generated by gene targeting using standard recombineering technology [19]. The targeting strategy is illustrated in S1A and S1B Fig. Genotyping of targeted ES cells was performed by Southern blot using the restriction enzyme BsoBI (S1C Fig.). Genotyping of Bcl2-ARE^flox/flox^ mice was performed by PCR (primers described in S1 Table). To test for germ-line recombination in Bcl2-ARE^flox/flox^ x mb1^cre^ mice, two different qPCR assays were designed: Assay A amplifies the intron 1—exon 2 junction of the Bcl2 gene. Assay B amplifies a region of 100 bp inside the loxP-flanked ARE sequence (S1D Fig.). The copy number and the ratio B/A were calculated as described in the extended materials and methods section (S1 Methods). Ratio B/A = 1 was scored by Bcl2-ARE^flox/flox^ x mb1^wt^ (flox/flox), ratio B/A = 0.5 was scored by Bcl2-ARE^flox/∆^ x mb1^wt^ mice, which contained one recombined allele (flox/∆). Ratio B/A = 0 was obtained when the Bcl2 ARE sequence was undetectable in the qPCR assay B (Bcl2-ARE^flox/flox^ x mb1^cre^, called ∆/∆ in the figures) (S1E Fig.).

### Mouse strains and animal procedures

The mouse strains used in this study are: C57BL/6, B6.SJL (B6.SJL-Ptprca Pep3b/BoyJ), FLPe-tg (mice from Dr. F. Stewart backcrossed into the C57BL/6 background), Bcl2-ARE^flox/flox^ (Bcl2^tm1Tnr^), mb1^cre^ (CD79a^tm1(cre)Reth^) and Bcl2.36 (C57BL/6-Tg(BCL2)36Wehi/J).

Bone marrow (BM) chimeras were generated using BM cells from B6.SJL, mb1^cre^ and Bcl2-ARE^flox/flox^ x mb1^cre^ mice that were harvested and mixed in a 1:1 ratio as follows: Control group—Cells from B6.SJL and mb1^cre^ mice; and ARE^∆/∆^ group—Cells from B6.SJL and Bcl2-ARE^flox/flox^ x mb1^cre^ mice. The mixture of BM cells was intravenously injected into lethally irradiated B6.SJL mice (10 Gy) (3x10^6 cells per mouse). After 8 weeks, cell reconstitution was tested by flow cytometry using specific antibodies against CD19, CD3 and CD45.2 cell surface markers. Peripheral immune tissues were analysed 12 weeks after bone marrow (BM) injection.

### Flow cytometry

Analysis of B and T cell populations in the blood, bone marrow, spleen and peripheral lymph nodes (LNs) was performed using specific antibodies (see S2 Table). DAPI was added to test cell viability. For intracellular staining of Bcl2 protein, the BD Cytofix/Cytoperm Fixation/Permeabilization Solution Kit (BD Biosciences) was used as indicated by the manufactures.

### Cell culture and Western blot

B cells from inguinal, brachial and axillary lymph nodes, or from spleen, were isolated by negative selection using the B Cell Isolation Kit from Miltenyi Biotec. B cells were cultured in RPMI 1640 Medium (Dutch Modification) (Life Technologies) supplemented with 5% FCS, antibiotics, 2 mM L-glutamine, 5 μM β-mercaptoethanol and 1 mM sodium pyruvate. In some experiments LPS (10 μg/ml, *E. coli* O127:B8 Sigma Aldrich) was used for cell stimulation.

Total cell extracts were prepared by incubating cells in RIPA buffer (50 mM Tris-HCl, pH 7.4, 150 mM NaCl, 1% NP-40, 0.1% SDS and 0.5% sodium deoxycholate) supplemented with protease inhibitors (Protease inhibitor cocktail 3, Cat. No. p8340, Sigma). After 15 minutes at 4°C, cell extracts were centrifuged and protein concentration in the supernatant was measured using a BCA protein assay (Pierce). 10% polyacrylamide-SDS gels were loaded with the indicated amount of protein extracts (10–20 μg per lane). Proteins were transferred to nitrocellulose membranes, and AUF-1 and tubulin proteins were detected with specific primary antibodies (see S2 Table). Blots were subsequently incubated with specific HRP-conjugated secondary antibodies and detected by enhanced chemiluminescence (Amersham Pharmacia Biotech).

### RNA extraction and qPCR

Total RNA from purified cells was isolated using TRIzol (LifeTech). 1 μg of RNA was reverse-transcribed to perform qPCR assays using Platinum Quantitative PCR SuperMix-UDG or SYBR Green PCR Master Mix (Life Technologies). The list of primers and Taqman probes used for these experiments are described in S1 Table. Quantification of Bcl2 gene expression was calculated by the ΔΔCT (comparative threshold cycle) method, following manufacturer’s instructions. Relative quantification of mRNA levels was determined using house-keeping genes (Actb and Hprt).

### RNA immunoprecitation

Analysis of HuR and AUF1 protein interaction with Bcl2 mRNA was tested in splenic B cells. Total protein extracts were obtained after cell lysis using RIPA buffer containing protease and RNase inhibitors (40 U/ml of RNase Out, Life Tech). Protein A/G magnetic beads (Qiagen) were coupled to 2 μg of antibody against AUF1 or HuR. After 1 hour incubation at room temperature, beads were washed with NET-2 buffer (50 mM Tris-HCl pH 7.5, 500 mM NaCl, 0.05% NP-40 and 1 mM MgCl_2_). Then, 250 μg of protein extracts were added to the beads and immunoprecipitation was carried out at 4°C, overnight. Beads were washed five times before protein was digested with 0.5 mg/ml of proteinase K in NET-2 buffer containing 0.1% SDS. After 15 minutes at 55°C, RNA was isolated from the supernatant by adding an equal volume of phenol: chloroform: isoamyl alcohol (25:24:1, Sigma, P3803). After mixing, samples were centrifuged to separate the hydrophilic and hydrophobic phases. RNA in the aqueous phase was precipitated using 0.1 volumes of 3 M NaOAc, 0.3 volumes of 1 mM EDTA and 2.5 volumes of 100% ethanol. Reverse-transcription of RNA into cDNA and qPCR assays were performed as described above. RNA immunoprecipitations using a mouse IgG_1_ antibody or a rabbit IgG antibody were performed in parallel as negative controls (see S2 Table for details about antibodies). Data from RNA immunoprecipitation assays are shown as fold enrichment relative to the negative controls. In all experiments, samples from two or three biological replicates were processed in parallel to determine biological variability. Data are shown as Mean + SD.

### Individual-nucleotide resolution Cross-Linking and ImmunoPrecipitation (iCLIP)

iCLIP experiments were performed as described [20]. Briefly, HuR-RNA interaction in intact splenic B cells was preserved by UV-crosslinking (150 mJ/cm^2^, Stratalinker 2400). Then, cells were washed with ice-cold PBS and lysed in RIPA buffer. After sonication (3 times), cell lysates were centrifuged and the supernatant digested with RNase I (0.167 U/ml, low RNase; or 6.67 U/ml, high-RNase) for 3 minutes at 37°C. Immunoprecipitation of RNA-protein complexes was performed following a similar protocol to the one performed for RNA immunoprecipitation (described above). Protein G dynabeads were coupled with 2 μg of antibody against HuR. After washing the beads with RIPA buffer, the cell extracts were added. Immunoprecipitation was performed overnight at 4°C. Cell extracts from HuR KO B cells were used as a negative control. Then, beads were washed twice with a high-salt buffer (50 mM Tris-HCl pH 7.4, 1 M NaCl, 1 mM EDTA, 1% NP-40, 0.1% SDS and 0.5% sodium deoxycholate) and once with PNK washing buffer (20 mM Tris-HCl pH 7.4, 10 mM MgCl_2_, 0.2% Tween-20). 1/10 of the RNA-protein complexes were labelled with ^32^P-ATP, whereas an RNA Linker was ligated to the rest of the sample after RNA dephosphorylation. Samples were then loaded in 10% SDS-PAGE gels and transferred to nitrocellulose membranes and RNA-protein complexes were visualised by autoradiography. RNA was isolated from the nitrocellulose membrane by incubating the fragment with 10 μl of proteinase K (Roche, 03115828001) in 200 μl of PK buffer (100 mM This-Cl pH 7.5, 50 mM NaCl and 10 mM EDTA). After 10 minutes at 37°C, 200 μl of PK buffer containing 7 M urea was added. Protein digestion was carried out for additional 20 minutes at 37°C. RNA was isolated by adding 1 volume of phenol/CHCl3 (Ambion, 9722) followed by ethanol precipitation as described previously. Reverse transcription to cDNA was performed using RCLIP primers and SuperScript III reverse transcriptase (Life Tech). cDNA was purified after running the sample in a 6% TBE-urea gel. Then, cDNA was extracted from the gel, circularised and amplified by PCR using Solexa P5/P7 primers. RCLIP primers are designed to add a nucleotide bar code with three known bases and four random nucleotides in the 5′ end of the cDNA molecule (called the 5′ barcode). The RNA linker introduces two known bases (AT) to the 3′end. This allowed us to multiplex samples for Illumina sequencing, to identify duplicated PCR reads and to quantify unique read counts mapped to the same genomic location. cDNA libraries were sequenced using Illumina GAIIX (40 bases and single read). Four samples were multiplexed per lane. cDNA libraries from three independent experiments were prepared and analysed.

### Bioinformatics and statistical analysis

Sequencing analysis of the iCLIP data was performed as previously described [20]. Briefly, sequencing raw data was demultiplexed and mapped against the mouse genome annotation mm9. Demultiplexing involved identification of the three known nucleotides of the 5’ barcode, and annotation of unique cDNA counts was performed after removing PCR duplicates that share the same four random bases of the 5’ barcode. Barcodes and any remaining Illumina adaptor sequence were trimmed out before mapping using Bowtie. Quality analysis of sequencing data was done using FASTQ and iCount pipeline. Once annotation of unique cDNA counts is completed, nucleotide-1 (the nucleotide bound by HuR) is annotated and used for data visualization in the UCSC genome viewer. Only data related to the Bcl2 locus is presented in this manuscript.

Phylo-VISTA [21] was used for phylogenetic analysis of Bcl2 exon 2 from human and mouse and Bcl2-ARE sequence alignment. Prediction of the interaction of RNA binding proteins to the Bcl2-ARE sequence was performed using catRAPID omics. Z-scoring is calculated as described [22]. The proteins considered for further analysis are those that have a described RNA-binding domain which binds to any of the RNA motifs predicted within the Bcl2-ARE rich sequence.

Mann-Whitney tests were performed for statistical analysis of the data.

### Ethics statement

All animal procedures were performed following authorization from the Babraham Institute Animal Welfare, Experimentation and Ethics Committee (AWEEC) and the UK Home Office.

## Results

### Deletion of the Bcl2 ARE-rich sequence in B cells reduces protein expression by decreasing mRNA stability and abundance

**Fig 1:**
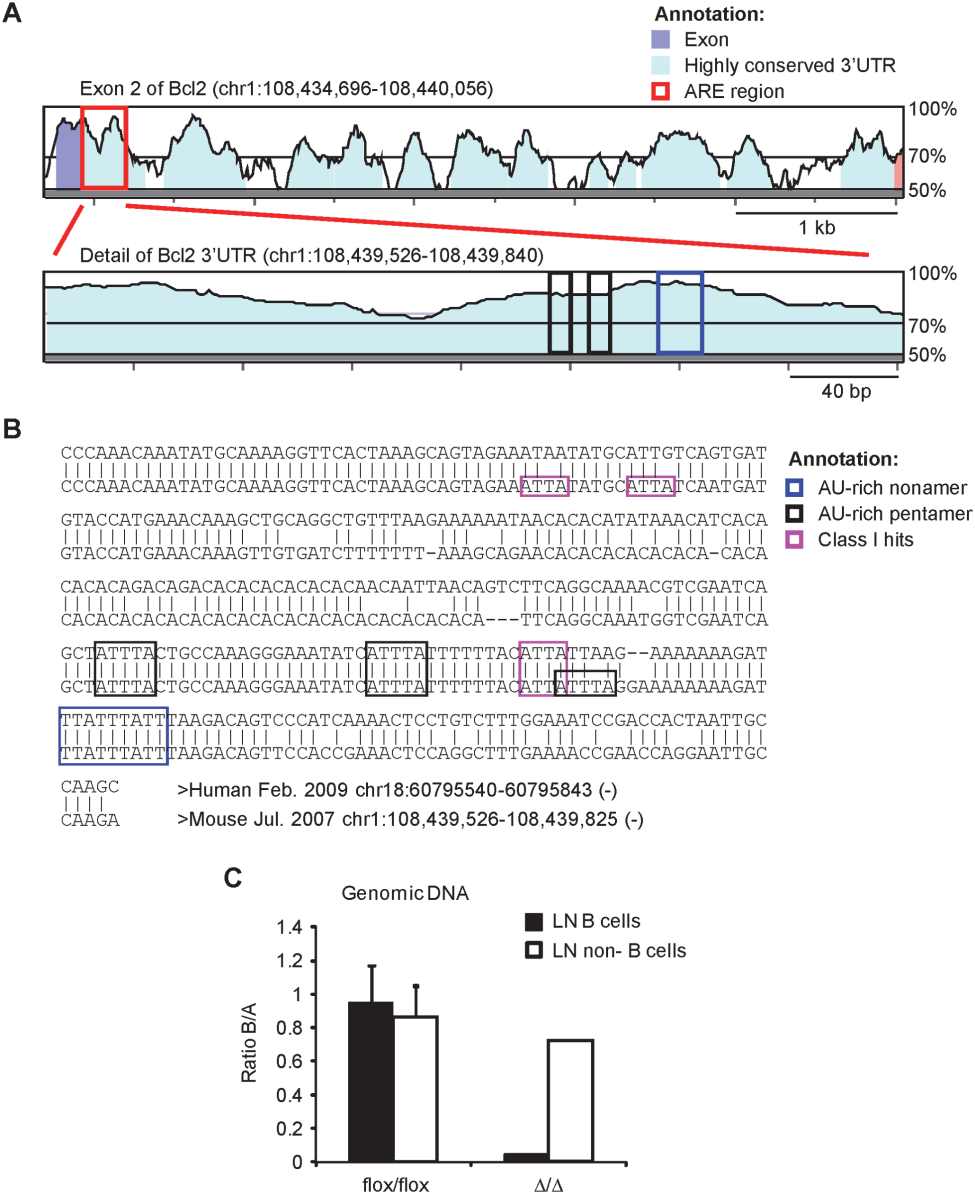
Description of the Bcl2 ARE-rich sequence removed in Bcl2-ARE^∆/∆^ B cells. **A**, Phylogenetic analysis of the exon 2 of Bcl2 (Phylo-VISTA analysis). **B**, Alignment of the human and mouse Bcl2 ARE-rich sequence that is deleted from the wild type allele in Bcl2-ARE^∆/∆^ (∆/∆) B cells (Bcl2-ARE^flox/flox^ x mb1^cre^ mice). **C**, Genomic analysis of Bcl2 alleles in B cells and non-B cells from Bcl2-ARE^flox/flox^ x mb1^wt^ (flox/flox) and Bcl2-ARE^flox/flox^ x mb1^cre^ (∆/∆) mice. CD19^+^ LN B cells were isolated by negative selection using magnetic beads. Positively labelled cells during the isolation procedure were processed as LN non-B cells. Genomic DNA was isolated and used for genotyping using two qPCR assays. Assay A assessed for DNA abundance, and assay B detected the loxP- flanked ARE. The ratio between assay B and assay A (Ratio B/A) is shown.

Analysis of murine Bcl2 3’UTR sequence shows high evolutionary conservation of the first 300 nucleotides located after the stop codon (>85% base conservation) as previously reported [10]. This region, (called Bcl2 ARE-rich sequence throughout the paper), contains one AU nonamer (UUAUUUAUU) and two AU pentamers (AUUUA), and they are all conserved in human (Fig. 1A and 1B). *In silico* analysis of the Bcl2 ARE-rich sequence using CatRAPID omics predicted the binding of several AUBPs, including Elav-like proteins, Tis11d and AUF-1, mainly through these conserved AREs (S3 Table).

To functionally characterise the importance of the Bcl2 ARE-rich sequence for Bcl2 expression in primary cells, we used gene targeting to generate the Bcl2-ARE^flox/flox^ mice, in which the Bcl2 alleles have been modified to introduce loxP sites flanking the Bcl2 ARE-rich sequence (S1A, S1B and S1C Fig.). After crossing Bcl2-ARE^flox/flox^ mice with mb1^cre^ mice, conditional deletion of the Bcl2 ARE-rich sequence was achieved in primary B cells by Cre-Lox recombination. In order to test cell-specific genomic deletion in Bcl2-ARE^flox/flox^ x m1^cre^ (∆/∆) mice, we isolated DNA from lymph nodes (LN) B cells and from the non-B cell fraction recovered after B cell depletion to perform qPCR assays using specific primers, that amplify the intron 1—exon 2 junction of the Bcl2 gene or a 100 bp region inside the loxP-flanked Bcl2 ARE-rich sequence (S1D Fig.). Deletion of the Bcl2 ARE-rich sequence was completed and restricted to B cells (Fig. 1C and S1E).

**Fig 2:**
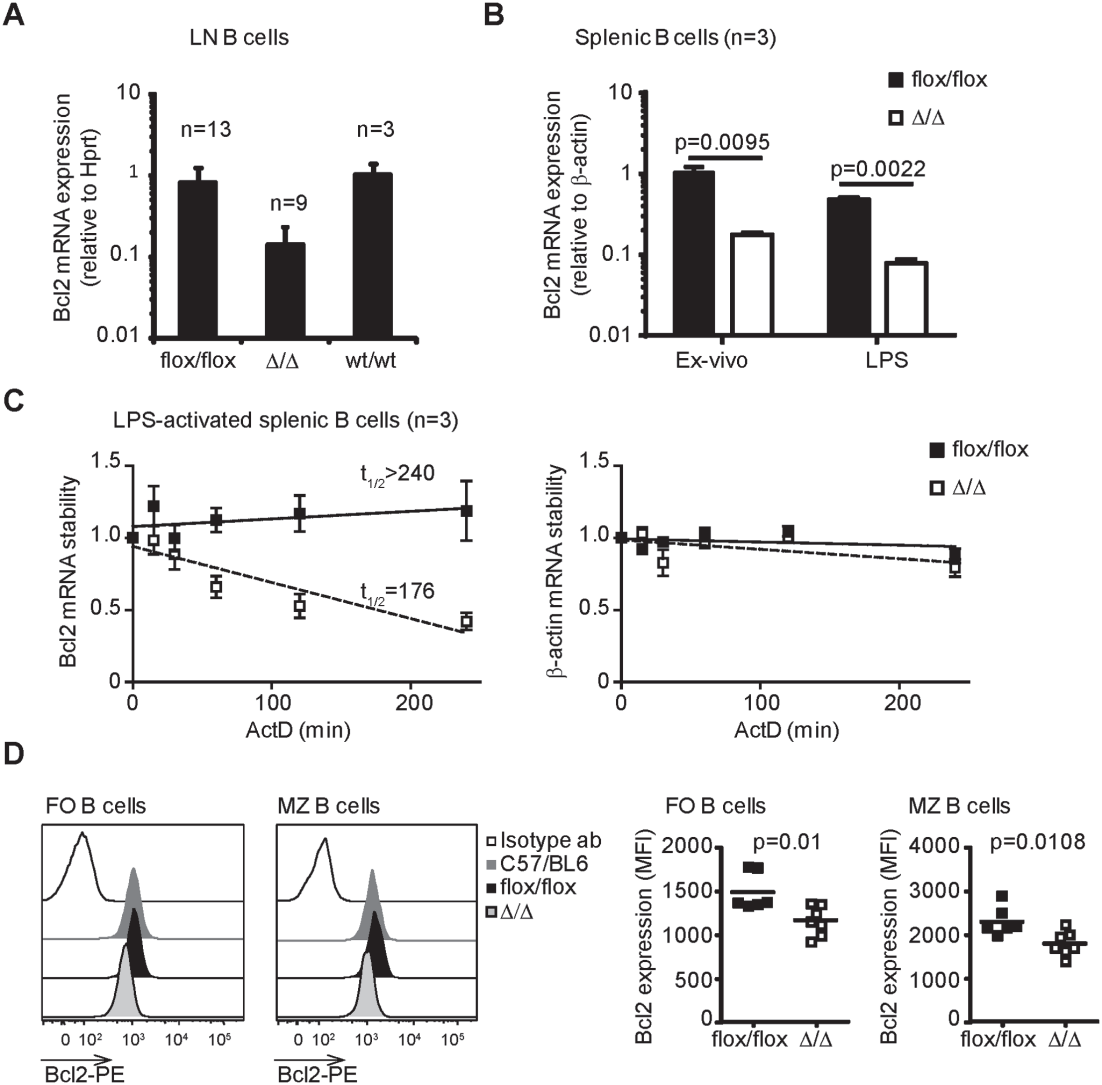
Bcl2 mRNA stability and protein expression are diminished in the absence of the Bcl2 ARE-rich sequence. **A**, qPCR analysis of Bcl2 mRNA levels in LN B cells from C57BL/6 (wt/wt), Bcl2-ARE^flox/flox^ x mb1^wt^ (flox/flox) and Bcl2-ARE^flox/flox^ x mb1^cre^ (∆/∆) mice. **B**, Relative quantification of Bcl2 mRNA in splenic B cells before and after stimulation with LPS (10 μg/ml) for 24 hours. β-actin was used as reference gene. n = 3 mice per genotype. **C**, Analysis of Bcl2 mRNA stability in splenic B cells from Bcl2-ARE^flox/flox^ x mb1^wt^ (flox/flox) and Bcl2-ARE^flox/flox^ x mb1^cre^ (∆/∆) mice. Splenic B cells were treated with LPS (10 μg/ml) for 24 hours before adding ActD (5 μg/ml). Cells were collected after 0, 15, 30, 60, 120 and 240 minutes and total RNA was extracted using TriZol. mRNA levels of Bcl2 and β-actin were assessed by qPCR. Data are shown as mRNA abundance relative to time 0. Data from one of the two independent experiments performed are shown. In both experiments, cells from three mice per genotype were processed independently to assess biological variability. Data are shown as the mean value + SD. **D**, flow cytometry analysis of Bcl2 protein expression in FO B cells (CD19^+^ CD93^-^ CD21^+^ CD23^+^ cells) and MZ B cells (CD19^+^ CD93^-^ CD21^+^ CD23^low^) from spleens of C57BL/6, Bcl2-ARE^flox/flox^ x mb1^wt^ (flox/flox) and Bcl2-ARE^flox/flox^ x mb1^cre^ (∆/∆) mice. Protein quantification is shown as median fluorescence intensity (MFI) (n = 6 mice per genotype). A non-parametric t test was performed for statistical analysis of the data in Fig. 2B and 2D. P values are shown.

Next, we analysed whether deletion of the Bcl2 ARE-rich sequence affected to the total amount of Bcl2 mRNA in B cells. The presence of loxP sites may affect mRNA levels if they interfere with regulatory elements [23]. Thus, we first tested whether insertion of the loxP sites flanking the Bcl2 ARE-rich sequence had any effects in Bcl2 mRNA levels. Analysis by qPCR showed that LN B cells from C57BL/6 and Bcl2-ARE^flox/flox^ mice had similar expression of Bcl2 mRNA relative to Hprt, suggesting that insertion of the loxP sites did not affect Bcl2 mRNA abundance (Fig. 2A). On the contrary, B cells from spleen or LNs of Bcl2-ARE^flox/flox^ x mb1^cre^ (∆/∆) mice had a five-fold reduction in Bcl2 mRNA levels compared to B cells from control Bcl2-ARE^flox/flox^ (flox/flox) mice. A similar reduction was observed after B cell stimulation with LPS. Altogether, Bcl2 ARE-rich sequence deletion reduced the amount of Bcl2 mRNA in B cells independently of the cell activation status (Fig. 2B).

Regulation of mRNA stability is a major factor that determines mRNA levels within the cell. In order to understand whether the Bcl2 ARE-rich sequence confers stability to Bcl2 mRNA, we analysed the Bcl2 mRNA levels present in splenic B cells at several time points after blocking transcription with Actinomycin D. These experiments showed that Bcl2 mRNA was very stable in control Bcl2-ARE^flox/flox^ B cells (t_1/2_ >240 min), but its half-life was significantly reduced in Bcl2-ARE^∆/∆^ B cells (t_1/2_ = 176 min) (Fig. 2C). Analysis of the mRNA levels of β-actin as control shows no change in stability irrespective of the cell genotype. Thus, deletion of the Bcl2 ARE-rich sequence led to the destabilization of Bcl2 mRNA.

Next, we measured Bcl2 protein expression by flow cytometry (Fig. 2D). B cells from C57BL/6 and Bcl2-ARE^flox/flox^ mice showed no difference in Bcl2 protein abundance, suggesting that loxP insertion flanking the Bcl2 ARE-rich sequence did not affect Bcl2 mRNA translation. By contrast, the amount of Bcl2 protein in Bcl2-ARE^∆/∆^ cells compared to control Bcl2-ARE^flox/flox^ cells was reduced by 30%. This reduction was observed both in follicular (FO) B cells and marginal zone (MZ) B cells, two subsets of mature B cells present in the spleen. Bcl2 protein abundance was similar in CD19^-^ cells and in CD4^+^ cells irrespective of the genotype, indicating that Bcl2 protein levels are selectively reduced in B cells after deletion of the Bcl2 ARE-rich sequence.

### Bcl2-ARE^flox/flox^ x mb1^cre^ mice have a reduced number of transitional and FO B cells

It has been previously shown that Bcl2 is required for the maintenance of mature B cells *in vivo* [6,7]. In order to understand whether deletion of the Bcl2 ARE-rich sequence affected B cell development or homeostasis, we analysed by flow cytometry the different B cell subsets present in the bone marrow, spleen and lymph nodes from Bcl2-ARE^flox/flox^ x mb1^cre^ mice.

**Fig 3:**
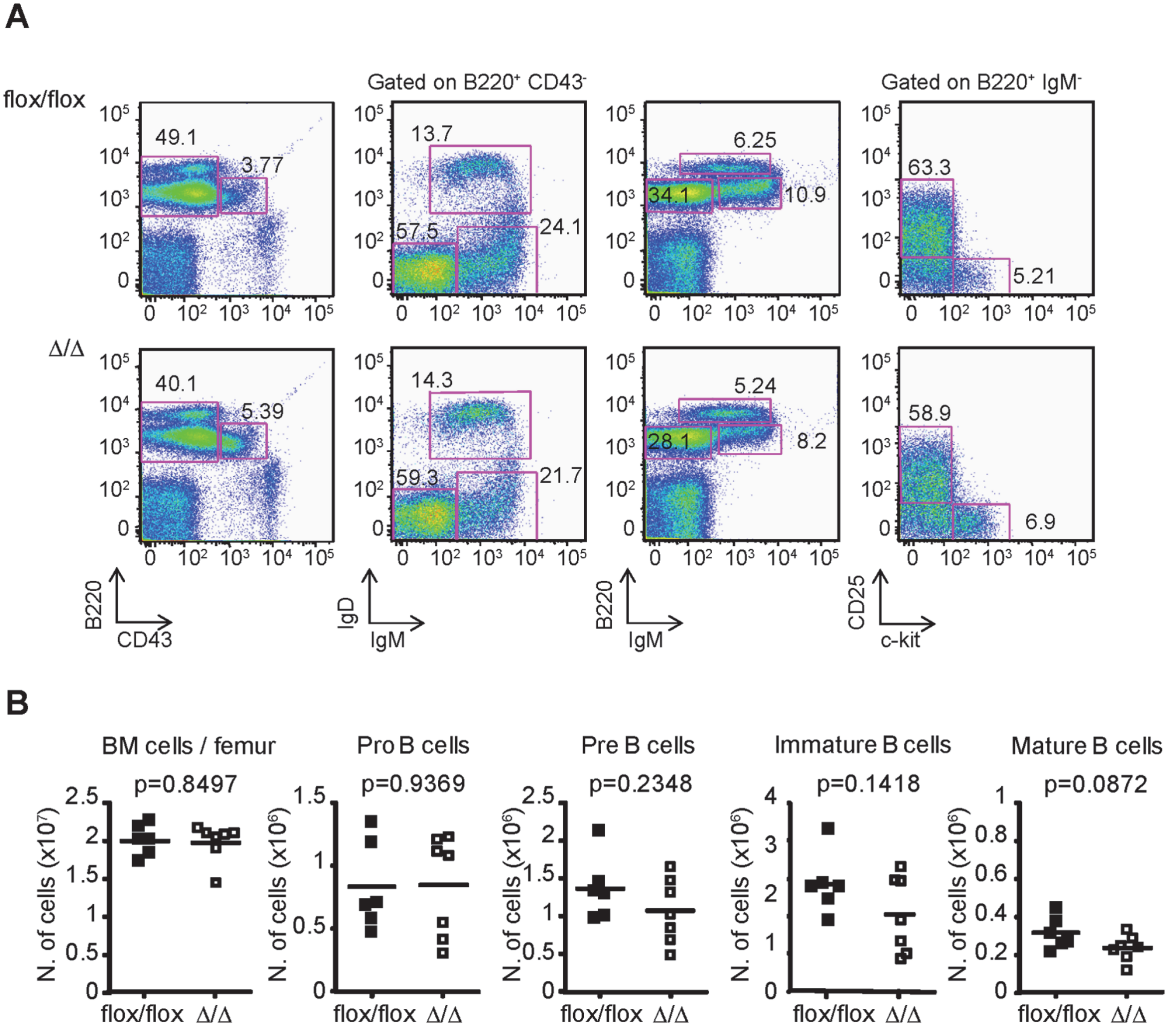
B cell development in the bone marrow is normal in Bcl2-ARE^flox/flox^ x mb1^cre^ mice. **A**, Flow Cytometry analysis of pro-B cells (B220^+^ IgM^-^ IgD^-^ c-kit^+^ CD25^-^ cells), pre-B cells (B220^+^ IgM^-^ IgD^-^ c-kit^-^ CD25^+^ cells), immature-B cells (B220^+^ CD43^-^ IgM^+^ IgD^-^ cells) and mature-B cells (B220^high^ CD43^-^ IgM^+^ IgD^+^ cells) from the bone marrow of Bcl2-ARE^flox/flox^ x mb1^wt^ (flox/flox) and Bcl2-ARE^flox/flox^ x mb1^cre^ (∆/∆) mice. N = 6–7 mice per genotype. The mean percentage of cells in each gate is shown within the plots. **B**, Quantification of total number of pro-, pre-, immature- and mature-B cells. A Mann-Whitney test was performed for statistical analysis of the data. P values are shown for each cell population.

Quantitation of the proportion and the number of pro-B cells (B220^+^ IgM^-^ IgD^-^ c-kit^+^ CD25^-^), pre-B cells (B220^+^ IgM^-^ IgD^-^ c-kit^-^ CD25^+^), immature B cells (B220^+^ CD43^-^ IgM^+^ IgD^-^) and recirculating B cells (B220^high^ CD43^-^ IgM^+^ IgD^+^) in the bone marrow showed no difference in any of these cell populations in Bcl2-ARE^flox/flox^ x mb1^cre^ (∆/∆) mice compared to Bcl2-ARE^flox/flox^ x mb1^wt^ (flox/flox) mice (Fig. 3).

**Fig 4:**
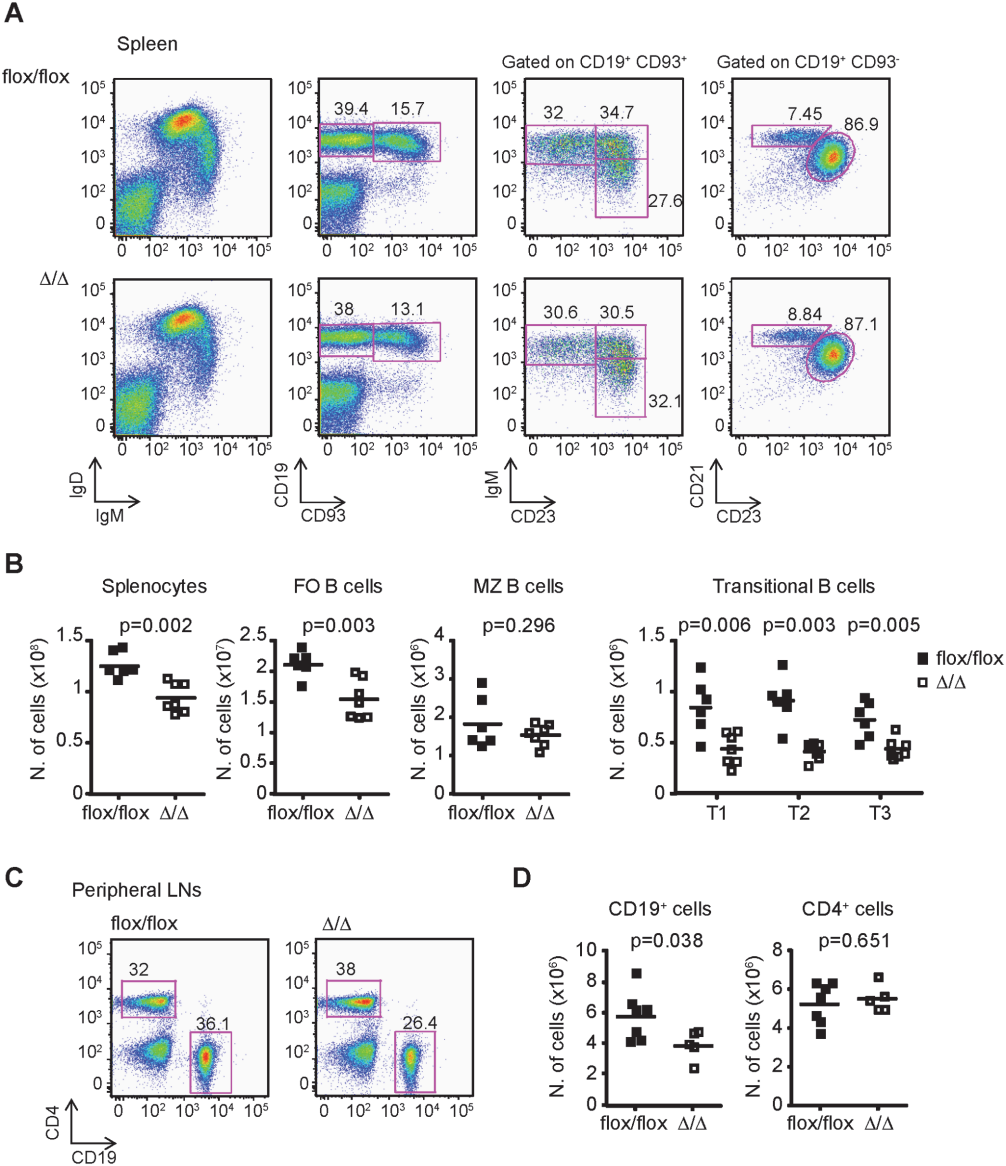
Bcl2-ARE^flox/flox^ x mb1^cre^ mice have a reduced numbers of FO B cells in the periphery. **A**, Flow cytometry analysis of FO B cells (CD19^+^ CD93^-^ CD23^+^ CD21^+^ cells), MZ B cells (CD19^+^ CD93^-^ CD23^low^ CD21^high^ cells) and transitional B cells (T1: CD19^+^ CD93^+^ IgM^+^ CD23^-^ cells; T2: CD19^+^ CD93^+^ IgM^+^ CD23^+^ cells; and T3: CD19^+^ CD93^+^ IgM^low^ CD23^+^ cells cells) in spleens from Bcl2-ARE^flox/flox^ x mb1^wt^ (flox/flox) and Bcl2-ARE^flox/flox^ x mb1^cre^ (∆/∆) mice. N = 6/7 mice per genotype. The mean percentage of cells in each gate is shown in each representative plot. **B**, Quantification of the number of total splenocytes, FO B cells, MZ B cells, and transitional B cells, defined as described above. **C**, Analysis of CD19^+^ B cells and CD4^+^ T cells in peripheral LN (inguinal, brachial and axillary LNs were pooled together for the analysis). The mean percentage of cells in each gate is shown in the plots. N = 5–7 mice per genotype. **D**, Quantitation of the number of CD19^+^ B cells and CD4^+^ T cells. A Mann-Whitney test was performed for statistical analysis of the data. P values are shown for each cell population.

Flow cytometry analysis of spleens showed a normal proportion of all B cell sub-populations defined as: follicular B cells (FO: CD19^+^ CD93^-^ CD23^+^ CD21^+^), marginal B cells (MZ: CD19^+^ CD93^-^ CD23^low^ CD21^high^) and transitional B cells (T1: CD19^+^ CD93^+^ IgM^+^ CD23^-^; T2: CD19^+^ CD93^+^ IgM^+^ CD23^+^; and T3: CD19^+^ CD93^+^ IgM^low^ CD23^+^) (Fig. 4A). However, quantitation of the numbers of transitional and FO B cells showed that these populations were reduced by 1.5 fold in Bcl2-ARE^flox/flox^ x mb1^cre^ (∆/∆) compared to Bcl2-ARE^flox/flox^ x mb1^wt^ (flox/flox) mice (Fig. 4B). By contrast, the numbers of MZ B cells were similar in both groups of mice.

The analysis of LNs showed a 1.5 fold reduction in the number of CD19^+^ B cells in Bcl2-ARE^flox/flox^ x mb1^cre^ (∆/∆) mice, whereas the number of CD4^+^ cells was similar when compared against control Bcl2-ARE^flox/flox^ x mb1^wt^ (flox/flox) mice (Fig. 4C and 4D). Altogether, phenotypic characterization of Bcl2-ARE^flox/flox^ x mb1^cre^ mice showed that the reduction in the expression of Bcl2 protein observed after deletion of the Bcl2 ARE-rich sequence has a moderate, but significant, impact on the number of FO B cells present in secondary lymphoid organs.

### Bcl2 ARE-rich sequence depletion reduces B cell survival

**Fig 5:**
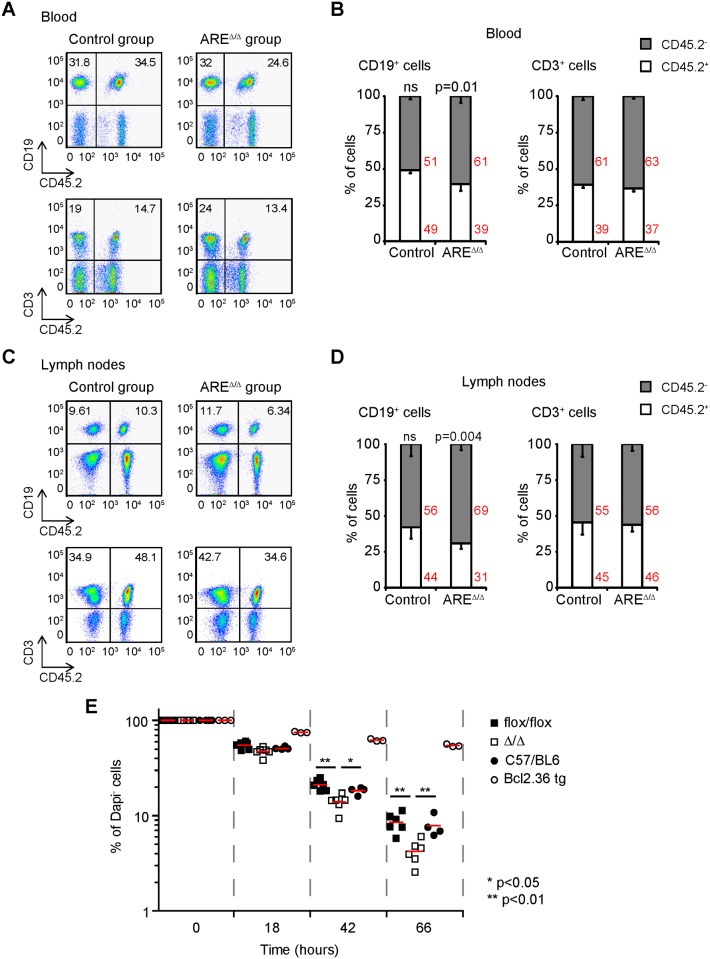
Loss of the Bcl2 ARE-rich sequence confers a competitive disadvantage to B cells. **A**, Flow cytometry analysis of competitive bone marrow chimeras. BM cells from B6.SJL and mb1^cre^ mice (control group) or B6.SJL and Bcl2-ARE^flox/flox^ x mb1^cre^ mice (ARE^∆/∆^ group) were mixed in a 1 to 1 ratio and injected into lethally irradiated B6.SJL mice. Blood samples were taken after eight weeks of BM reconstitution for flow cytometry using CD19, CD3 and CD45.2 as cell markers. **B**, Analysis of B and T cell proportions in BM chimera mice. A Mann-Whitney non parametric test was performed for statistical analysis of the data. P values are indicated. n = 8–9 mice per genotype. **C**, Analysis of B and T cells present in peripheral LNs of the mice described in A. Representative pseudo-colour dot plots are shown. **D**, Quantitation of B and T cell proportions in LNs. **E**, Survival analysis of LN B cells from C57BL/6, Eμ-bcl2–36, Bcl2-ARE^flox/flox^ x mb1^wt^ (flox/flox) and Bcl2-ARE^flox/flox^ x mb1^cre^ (∆/∆) mice. B cells were cultured *in vitro* in the absence of cytokines. Cell survival was tested at the indicated times by DAPI staining. A Mann-Whitney test was performed for statistical analysis of the data.

In order to test whether the reduced expression of Bcl2 affected the ability of Bcl2-ARE^∆/∆^ B cells to compete with wild type B cells *in vivo*, we reconstituted lethally irradiated B6.SJL mice with 1:1 mixtures of bone marrow cells from B6.SJL and mb1^cre^ mice (control group) or from B6.SJL and Bcl2-ARE^flox/flox^ x mb1^cre^ mice (ARE^∆/∆^ group). Eight weeks later, flow cytometry analysis of CD19^+^ cells present in the blood of mice from the control group showed equivalent proportions of CD45.1^+^ and CD45.2^+^ cells, indicating successful reconstitution at a 1:1 ratio (Fig. 5A and 5B). Analysis of the mice from the ARE^∆/∆^ group showed a significant 1.5 fold reduction in the proportion of Bcl2-ARE^∆/∆^ (CD45.2^+^) B cells compared to the proportion of B6.SJL (CD45.1^+^) cells (comparison of CD45.1^+^ vs CD45.2^+^ B cells within the ARE^∆/∆^ group) or to the proportion of mb1^cre^ (CD45.2^+^) cells (comparing the proportion of CD45.2^+^ cells present in the control group vs the ARE^∆/∆^ group) (Fig. 5A and 5B). Further analysis of the different B cell subsets in LN and spleen showed again a significant 1.5 fold reduction in the proportion of mature Bcl2-ARE^∆/∆^ (CD45.2^+^) B cells compared to B6.SJL (CD45.1^+^) B cells present in mice from the ARE^∆/∆^ group. On the other hand, the proportion of CD3^+^ cells and transitional B cells remained close to the 1:1 ratio (Fig. 5C, 5D and S2). In summary, these results suggested that FO and MZ mature B cells lacking the Bcl2-ARE rich sequence have a competitive disadvantage compared to wild-type cells.

Bcl2 expression promotes B cell survival *in vivo* and *in vitro* [6]. In order to test the viability of Bcl2 ARE^∆/∆^ B cells, we performed *in vitro* LN B cell cultures in the absence of cytokines or mitogens. At indicated time points, cell viability was tested by staining double-stranded DNA with DAPI. Bcl2-ARE^∆/∆^ B cells showed increased cell death compared to control B cells from C57BL/6, mb1^cre^ or Bcl2-ARE^flox/flox^ mice (Fig. 5E). On the contrary, Bcl2 over-expression (Bcl2.36tg B cells) prevented cell death as shown previously [24]. Taken together, diminished Bcl2 expression in B cells from Bcl2-ARE^flox/flox^ x mb1^cre^ mice reduces B cell survival.

### AUF1 and HuR bind to the Bcl2 ARE-rich sequence

**Fig 6:**
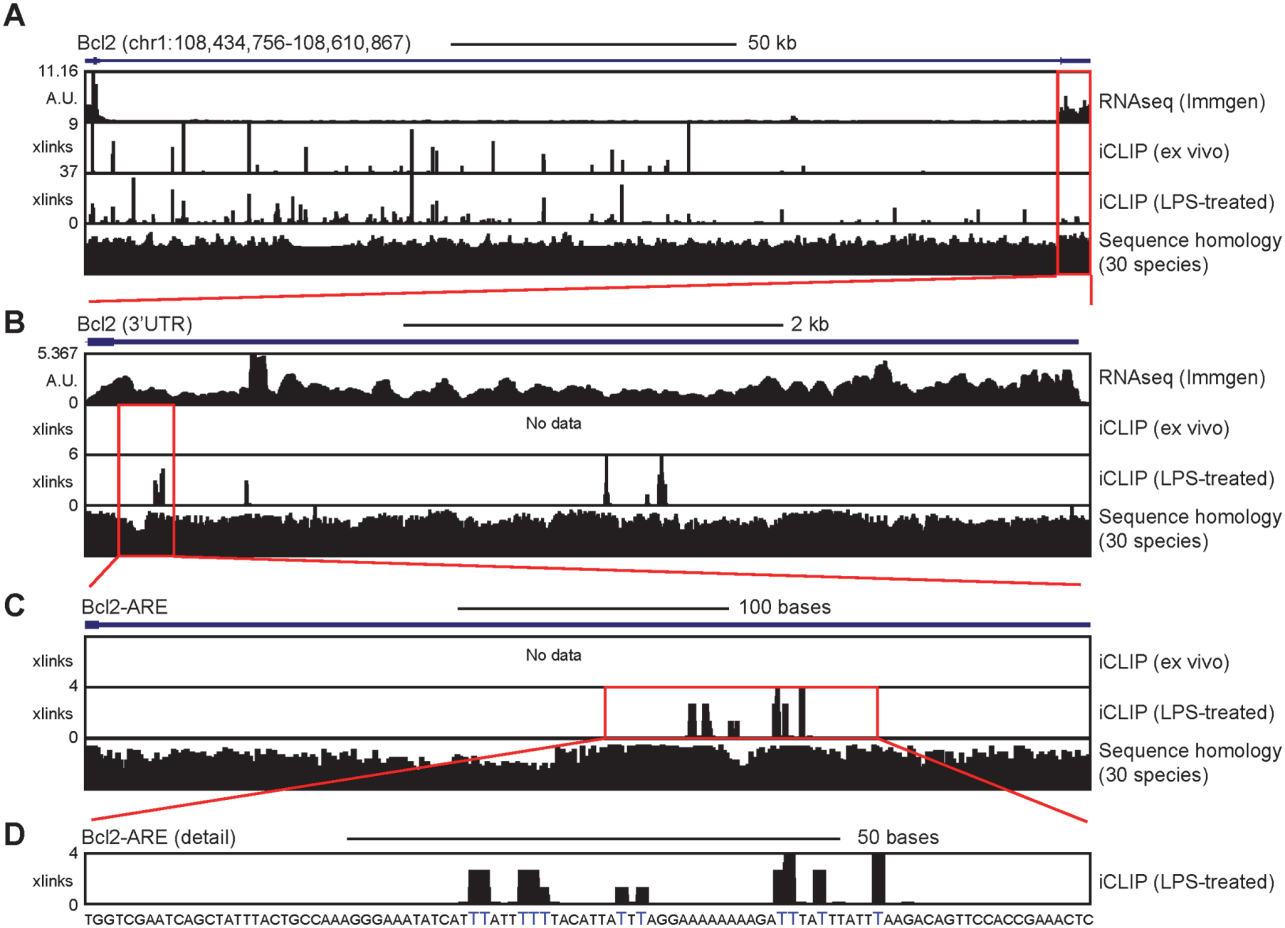
HuR only binds to the Bcl2 ARE-rich sequence after B cell activation. **A**, Genomic annotation of HuR- crosslinks across the Bcl2 locus. Representative data from iCLIP experiments (n = 3) performed with total cells extracts from freshly isolated B cells or LPS-activated B cells are shown. The number on the left hand of each genomic track refers to the maximum number of unique counts identified at a single nucleotide level (called cross(x)-links). Bcl2 expression data from CD19^+^ CD3^-^ splenic B cells (RNAseq from ImmGen.org) and sequence homology analysis across 30 species are shown in independent genomic tracks. **B**, Representation of HuR- crosslinks within the 3’UTR of Bcl2. **C**, Analysis of HuR binding to the Bcl2 ARE-rich sequence. **D**, Sequence analysis of HuR binding to the ARE. Single nucleotide interactions with HuR are coloured in blue.

Bcl2 mRNA stability and translation into protein is likely mediated by the RBPs that are bound to the Bcl2 ARE-rich sequence. Thus, we asked whether bioinformatics analysis of the nucleotide composition of the Bcl2 ARE-rich sequence could predict the interaction of different RBPs prior to validation by standard biochemical procedures. First, we performed a catRAPID omics analysis and positively sorted those predicted RBPs which have at least one characterised RNA-binding domain able to bind to AU-rich elements (S3 Table). Several splicing regulators were identified (i.e. Srsf2, Srsf9, Tia-1, Nova-1, Nova-2, Ptbp-1 and Pcbp22 (Hnrpe2)), but they are unlikely to regulate Bcl2 mRNA stability because they mostly bind to intronic regions [25–27]. HuR (ELAVL1) and AUF1 (HNRPD), which have been previously identified as protein regulators of Bcl2 mRNA stability, were also identified as putative RBPs in the catRAPID omics analysis [12,28]. HuR protein expression is induced after B cell activation with LPS (S3A Fig.). In order to validate the bioinformatics prediction, we analysed two publically available HuR PAR-CLIP datasets [29,30], but failed to undoubtedly characterised HuR binding to the 3’UTR of Bcl2 (S4A and S4B Fig.). HuR association with the Bcl2 3’UTR was not observed in the PAR-CLIP experiments performed in HeLa cells by Lebedeva et al [29]; whereas HuR was bound to six different locations across the Bcl2 3’UTR in the only PAR-CLIP experiment performed in HEK293 cells by Mukherjee et al [30]. Therefore, we performed HuR iCLIP experiments to map with single nucleotide resolution the interactions between HuR and Bcl2 mRNA in primary mouse B cells. We used HuR KO B cells to test the specificity of the antibody used for immunoprecipitation of the HuR:RNA complexes (S3B and S3C Fig.). iCLIP assays, performed using freshly isolated splenic B cells, failed to identify HuR binding to the Bcl2 ARE-rich sequence (Fig. 6, S4C and S4D). Furthermore, RIP assays also failed to detect co-immunoprecipitation of HuR and mature Bcl2 mRNA, suggesting that HuR is dispensable for the stabilization of mature Bcl2 mRNA in freshly isolated B cells (S4E Fig.). HuR was only found associated to the AU-rich elements present within the Bcl2 ARE-rich sequence when protein extracts from mitogen-activated B cells were used to perform RIP or iCLIP experiments. Taken together, HuR binding to the Bcl2 ARE-rich sequence is dynamic and depends on B cell activation.

**Fig 7:**
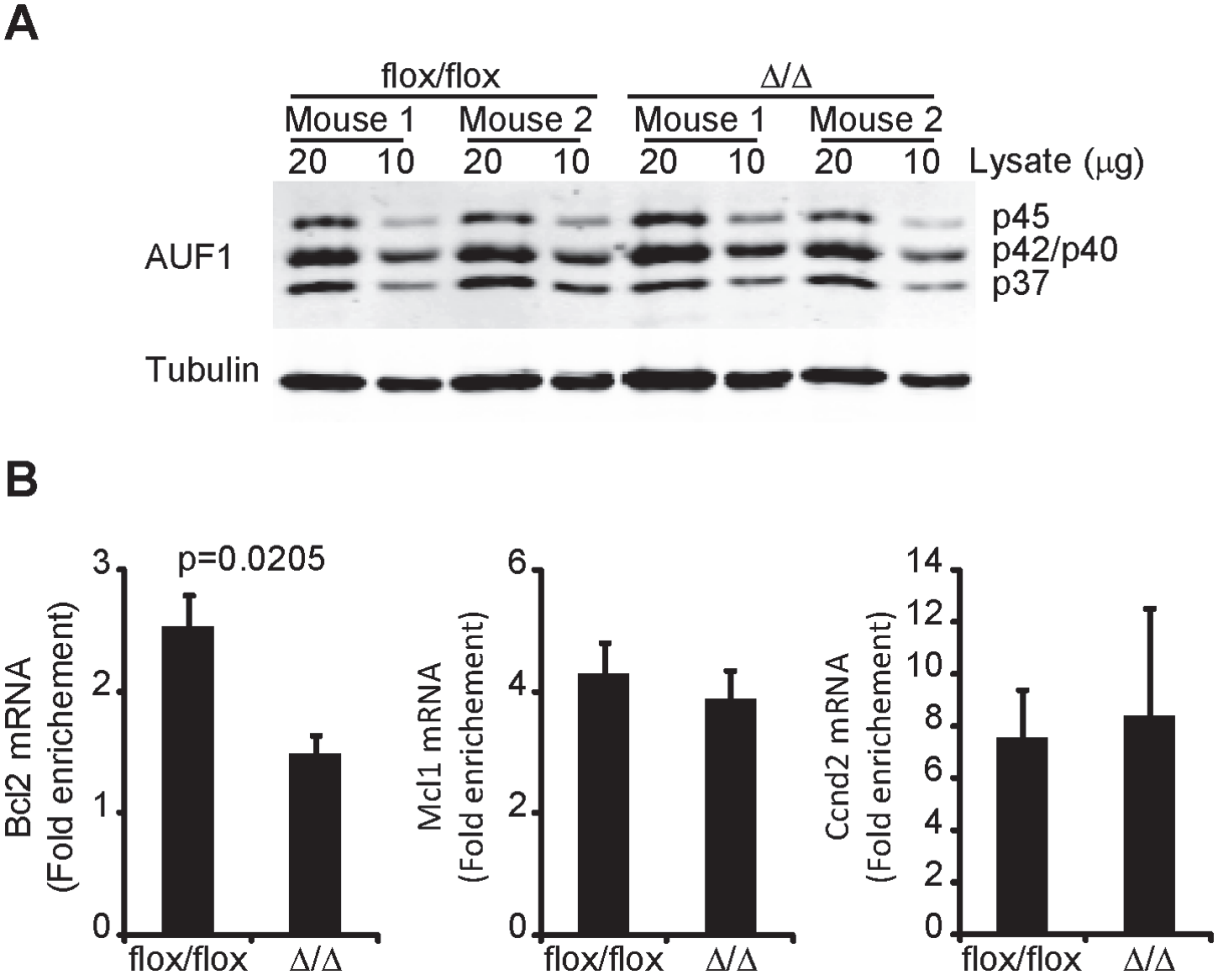
AUF1 binding to Bcl2 mRNA is reduced in the absence of the Bcl2 ARE-rich sequence. **A**, Analysis of AUF1 protein expression in splenic B cells from Bcl2-ARE^flox/flox^ x mb1^wt^ (flox/flox) and Bcl2-ARE^flox/flox^ x mb1^cre^ (∆/∆) mice. Two different amounts of total cell lysates (10 and 20 μg) were loaded in SDS-PAGE gels. AUF1 protein was detected by Western blot with tubulin used as loading control. **B**, AUF1 immunoprecipitation. Total cell extracts from splenic B cells from Bcl2-ARE^flox/flox^ x mb1^wt^ (flox/flox) and Bcl2-ARE^flox/flox^ x mb1^cre^ (∆/∆) mice were used for RIP experiments using a specific antibody against AUF1 or an isotype rabbit IgG antibody. Bcl2 mRNA was detected by qPCR. Mcl1 and Ccnd2 mRNAs were detected as control mRNAs of AUF1-RNA IP. Data are shown as mRNA fold enrichment (AUF1 IP / IgG IP). The data shown in this figure are from three independent experiments. In each experiment, splenic B cells from 2–3 mice per genotype were processed independently to assess biological variability. A Mann-Whitney test was performed for statistical analysis of the data.

CatRAPID omics analysis also predicted AUF1 binding to the Bcl2 ARE-rich sequence. Thus, we analysed AUF1 interaction with the Bcl2 ARE-rich sequence by RNA immunoprecipitation using total extracts from freshly isolated splenic B cells from Bcl2-ARE^flox/flox^ x mb1^wt^ (flox/flox) and Bcl2-ARE^flox/flox^ x mb1^cre^ (∆/∆) mice. First, we confirmed that AUF1 protein was equally abundant in lysates prepared from both cell genotypes (Fig. 7A). Then, we pulled down the AUF1:RNA complexes using a specific antibody against AUF1 and detected the presence of specific mRNAs in the immunoprecipitate by qPCR. Equivalent amounts of Ccnd2 mRNA and Mcl1 mRNA were immunoprecipitated in both genotypes. Bcl2 mRNA was also pulled down together with AUF1 when total protein extracts from wild-type mature B cells were used in the RIP assay. However, enrichment of Bcl2 mRNA was hardly detectable when protein extracts from Bcl2-ARE^∆/∆^ B cells were used, suggesting that the interaction between AUF1 and Bcl2 mRNA requires the Bcl2 ARE-rich sequence (Fig. 7B).

## Discussion

The binding of RBPs to the AREs present within the Bcl2 3’UTR modulates mRNA stability and Bcl2 protein expression within the cell [10]. Distinctive roles have been attributed to different RBPs, which can either promote or block Bcl2 mRNA degradation, but the overall importance of the AREs to control Bcl2 mRNA fate has remained unknown until now. In this study we provide insight into the function of the Bcl2 ARE-rich sequence as a promoter of Bcl2 mRNA stability. Primary B cells lacking the Bcl2 ARE-rich sequence have a decreased expression of Bcl2 protein as a consequence of a reduction in the half-life of the Bcl2 mRNA, which is diminished in abundance. Bcl2 expression is required for the maintenance of mature B cells in the periphery, but it is dispensable during B cell development, in which other members of the Bcl2-family, such as Mcl1, play an important role [6,7,31]. Reduced Bcl2 expression in Bcl2-ARE^flox/flox^ x mb1^cre^ mice negatively affects homeostasis of mature B cells, which are reduced in number in intact mice and show a competitive disadvantage in populating the peripheral lymphoid organs compared to wild type B cells.

This is one of the few studies that have investigated the physiological functions of the AREs *in vivo*. AREs are generally present in short-lived transcripts and are related with mRNA decay and reduced expression levels, although factors like the length of 3’UTR or the distance between the stop codon and the polyA signal may influence the stability of specific mRNAs [32]. Removal of the AREs present in the 3’UTR of TNF or GM-CSF has revealed an inhibitory role of these cis-acting sequences in mRNA stabilization and protein production [33,34]. Interestingly, our data suggest that the Bcl2 ARE-rich sequence in aggregate acts to stabilize the Bcl2 mRNA in mature B cells. Further characterization of the RBPs associated with the AREs is fundamental to understand the physiological functions of these regulatory elements. In this study we have used bioinformatics tools to predict possible interactions between RBPs and the Bcl2 ARE-rich sequence. Many of the RBPs identified as putative modulators of Bcl2 mRNA stability, including Nova-1/2 and three Elavl- proteins, are neuron-specific RBPs, which suggested that the biological relevance of these *in silico* predictions should be addressed by manipulating the expression of the relevant RBPs in a tissue dependent manner. Here we have validated the interaction of two RBPs, HuR (ELAVL1) and AUF1, that are both expressed in B lymphocytes and have been previously related to the stabilization of Bcl2 mRNA [12,35].

Conditional deletion of HuR in BM precursors is associated with a decreased expression of Bcl2 mRNA, which has been correlated with increased death of hematopoietic cells [18]. Our iCLIP data indicate that binding of HuR to the AREs is dynamic and varies depending on B cell activation. HuR fails to bind to the Bcl2 ARE-rich sequence in resting B cells suggesting that it is dispensable for Bcl2 mRNA stabilization in quiescent mature B cells. Much evidence in the literature has shown how post-translational modification of RBPs affects the stability and translation of their mRNA targets by altering the RNA binding or functional properties of the RBPs [36,37]. The best example in the context of the immune system comes from the study of the post-transcriptional regulatory mechanisms involved in TNF mRNA stabilization and translation. Like Bcl2, TNF mRNA has several AREs within the 3′UTR that regulate mRNA half-life. Treatment of macrophages with LPS enhances TNF production, not only by increasing mRNA transcription but, also, by promoting mRNA stabilization and translation. HuR and Tis11 compete to bind to the ARE of TNF mRNA. LPS-mediated activation of the p38 MAPK/MK2 pathway promotes Tis11 phosphorylation and displacement from the AREs that are now bound by HuR, which promotes transcript stabilization and translation [38]. Recently, Zekavati et al. showed that Tis11b binds to Bcl2 AREs [16] and our bioinformatics analysis identified Tis11d as a candidate RBP that may bind to the Bcl2 ARE-rich sequence. All members of the Tis11-family of proteins have highly conserved zinc-finger domains that bind to AREs [39] and may regulate Bcl2 mRNA destabilization and decay. Several RBPs with opposite functions may compete to bind to the Bcl2 ARE-rich sequence, and their affinity for AREs may change due to post-translational modifications [13]. The development of streptavidin-bound RNA aptamers specific to the Bcl2 ARE-rich sequence will allow to purify and quantify the RNP complexes bound to the Bcl2 mRNA [40].

We have also provided in this study several lines of evidence indicating that AUF1 binding to the mature Bcl2 mRNA is mediated by the Bcl2 ARE-rich sequence. This observation validates previous *in vitro* assays that point to AUF1 as a regulator of Bcl2 mRNA stability [28]. AUF1^-/-^ mice have reduced numbers of FO B cells in spleen [17], similar to the B cell phenotype observed in the Bcl2-ARE^flox/flox^ x mb1^cre^ (∆/∆) mice. Sadri et al. attribute this defect to a reduced cellular expression of pro-survival proteins, including Bcl2, Bcl-XL and A1, but they did not provide biochemical evidence of AUF1 binding to any of these target mRNAs. Here we show that AUF1 binds to the Bcl2 mRNA, which may contribute to B cell maintenance in the periphery.

AUF1 may be involved in the stabilization of Bcl2 mRNA, but we cannot exclude roles for other RBPs, like nucleolin, which may contribute to this effect in chronic lymphocytic leukemia cells [41]. Generation of the G-rich DNA aptamer AS1411, that targets nucleolin, has been proven effective in destabilising Bcl2 mRNA in MCF7 breast cancer cells [42]. Conditional deletion of the Bcl2 ARE-rich sequence provides a powerful tool in order to characterize the complex of RBPs that are associated to this regulatory sequence and further understand how Bcl2 mRNA stability is regulated in different cell types and under different stimulatory conditions. This knowledge is essential in order to develop further new therapeutic approaches for cancer treatment, in which mRNA destabilization can be promoted by targeting specific RBPs to decrease the expression of oncogenes, like Bcl2.

## Supporting Information

S1 Fig Generation of the Bcl2-ARE^flox/flox^ mice. **A**, Schematic representation of Bcl2 wild type allele, Bcl2 targeted allele and Bcl2-ARE^flox^ allele (ARE = AU-rich element; Flp = flippase recombinase; FRT = flippase recognition target; Neo = neomycin resistant gene; loxP = locus of X-over P1 sequence). **B**, DNA sequence present in the Bcl2-ARE^flox/flox^ allele. Recombination sites and extra sequences from the cloning vector are indicated. (ARE-rich sequence = italic text; loxP site = blue text; AU-pentamers = red text; FRT site = grey text; AU-nonamer = yellow text). **C**, Screening of targeted ES cells by Southern Blot using the restriction enzyme BsoBI. **D**, Summary of mouse genotyping strategy by qPCR. **E**, Representative analysis of qPCR assay A, that assesses DNA abundance, and qPCR assay B, that detects the loxP- flanked ARE. Mouse genotype was assessed after calculating the B/A ratio. Data from germline recombination in Bcl2-ARE^flox/flox^ x mb1^cre^ mice are shown.
(TIF)

S2 Fig Loss of the Bcl2 ARE-rich sequence confers a competitive disadvantage to B cells. Analysis of the proportions of the different subsets of B cells in the spleen of the competitive bone marrow chimeras described in Fig. 5. Cell populations are defined as: transitional T1 B cells (CD19^+^ CD93^+^ IgM^+^ CD23^-^ cells), transitional T2 B cells (CD19^+^ CD93^+^ IgM^+^ CD23^+^ cells), transitional T3 B cells (CD19^+^ CD93^+^ IgM^low^ CD23^+^ cells cells), FO B cells (CD19^+^ CD93^-^ CD23^+^ CD21^+^ cells) and MZ B cells (CD19^+^ CD93^-^ CD23^low^ CD21^high^ cells). A Mann-Whitney non parametric test was performed for statistical analysis of the data. P values are indicated. n = 8–9 mice per genotype.
(TIF)

S3 Fig Immunoprecipitation of HuR:RNA complexes. **A**, Analysis by Western Blot of HuR protein expression in freshly isolated splenic B cells and in B cells activated with LPS for 24 or 48 hours. β-actin is used as loading control **B**, Validation of HuR immunopreciptation. Splenic B cells from wild-type and HuR^flox/flox^ x mb1^cre^ mice were stimulated with LPS for 48h before isolation of the total protein extracts used in the immunoprecipitation assays. 2 μg of a mouse IgG_1_ against HuR (3A2 clone, Santa Cruz) or 2 μg of an isotype mouse IgG_1_ (MOPC21 clone, Sigma Aldrich) were used as indicated in Material and Methods. **C**, Representative x-ray film detecting radioactive labelled- HuR:RNA complexes. Total cell extracts from LPS-activated B cells irradiated with UV-light (150 mJ/cm^2^) were used to immunoprecipitate the HuR:RNA complexes after partial RNA digestion with RNase I. The same antibodies described in **B** were used for the immunoprecipitation and HuR:RNA complexes were detected after RNA labelling with ATP-gamma-32P. The dot line indicates the molecular weight of highly digested RNA molecules cross-linked to HuR. HuR:RNA complexes with approximately a molecular weight from 55 to 80 KDa (red box) were isolated for cDNA library preparation.
(TIF)

S4 Fig HuR only binds to the Bcl2 ARE-rich sequence after B cell activation. **A, B**, Analysis of HuR-Bcl2 mRNA interaction in HeLa and HEK293 cells. PAR-CLIP data from Lebedeva et al. (Mol. Cell. 2011 Aug 5;43(3)340–52) and Mukherjee et al. (Mol. Cell. 2011 Aug 5;43(3):327–39) was visualised using the UCSC genome browser and hg18 (**A**) and hg19 (**B**) respectively. Bcl2 ARE-rich sequence is indicated by a red box. **C**, Identification in primary B cells of HuR binding sites across the Bcl2 3’UTR. iCLIP data from three independent iCLIP experiments performed using protein extracts from freshly isolated B cells or LPS-activated B cells were visualised using the UCSC genome browser and mm9 genome annotation. Sum data of the three iCLIP experiments per condition are also shown. **D**, Mapped iCLIP data along the Actb gene is shown as experimental control. **E**, Validation of HuR-Bcl2 mRNA interaction by RNA immunoprecipitation assays. Total protein extracts from freshly isolated splenic B cells or cells treated with LPS for 48 hours were used for HuR:RNA immunoprecipitation using 2 μg of a mouse IgG_1_ against HuR (3A2 clone, Santa Cruz) or 2 μg of an isotype mouse IgG_1_ (MOPC21 clone, Sigma Aldrich) as negative control. Bcl2 mRNA associated to HuR was detected by qPCR. Data from two independent experiments are shown as mRNA fold enrichment relative to the IgG_1_ IP controls.
(TIF)

S1 Table List of primers and Taqman assays used for qPCR. (XLS)

S2 Table List of antibodies used for Flow cytometry, Western Blot and RNA-IP. (XLS)

S3 Table *In silico* analysis of Bcl2 ARE-rich sequence and its interaction with RBPs. *In silico* prediction of RBPs binding to the Bcl2 ARE-rich sequence (catRAPID omics).
(XLS)

S1 Methods Extended materials and methods related to the generation by recombineering and genotyping of Bcl2-ARE^flox/flox^ mice. (DOCX)
