# Supplementary figures and images for: Deletion of AU-Rich Elements within the Bcl2 3′UTR Reduces Protein Expression and B Cell Survival In Vivo
Source: PLoS One. 2015 Feb 13;10(2):e0116899. doi: 10.1371/journal.pone.0116899 (PMC4332480; doi:10.1371/journal.pone.0116899)

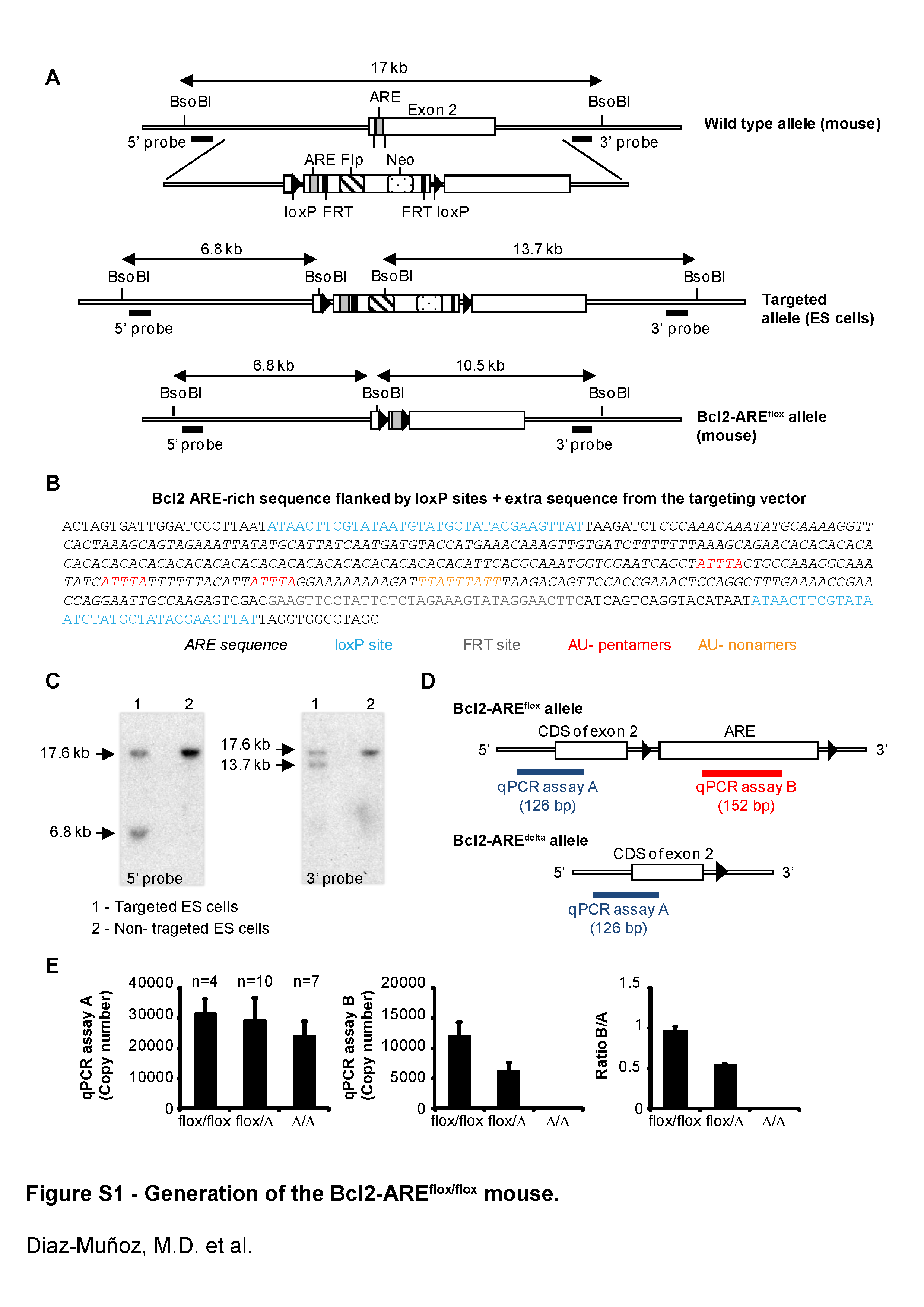

Supplement: S1 Fig — A, Schematic representation of Bcl2 wild type allele, Bcl2 targeted allele and Bcl2-AREflox allele (ARE = AU-rich element; Flp = flippase recombinase; FRT = flippase recognition target; Neo = neomycin resistant gene; loxP = locus of X-over P1 sequence). B, DNA sequence present in the Bcl2-AREflox/flox allele. Recombination sites and extra sequences from the cloning vector are indicated. (ARE-rich sequence = italic text; loxP site = blue text; AU-pentamers = red text; FRT site = grey text; AU-nonamer = yellow text). C, Screening of targeted ES cells by Southern Blot using the restriction enzyme BsoBI. D, Summary of mouse genotyping strategy by qPCR. E, Representative analysis of qPCR assay A, that assesses DNA abundance, and qPCR assay B, that detects the loxP- flanked ARE. Mouse genotype was assessed after calculating the B/A ratio. Data from germline recombination in Bcl2-AREflox/flox x mb1cre mice are shown. (TIF) [file pone.0116899.s001.tif]

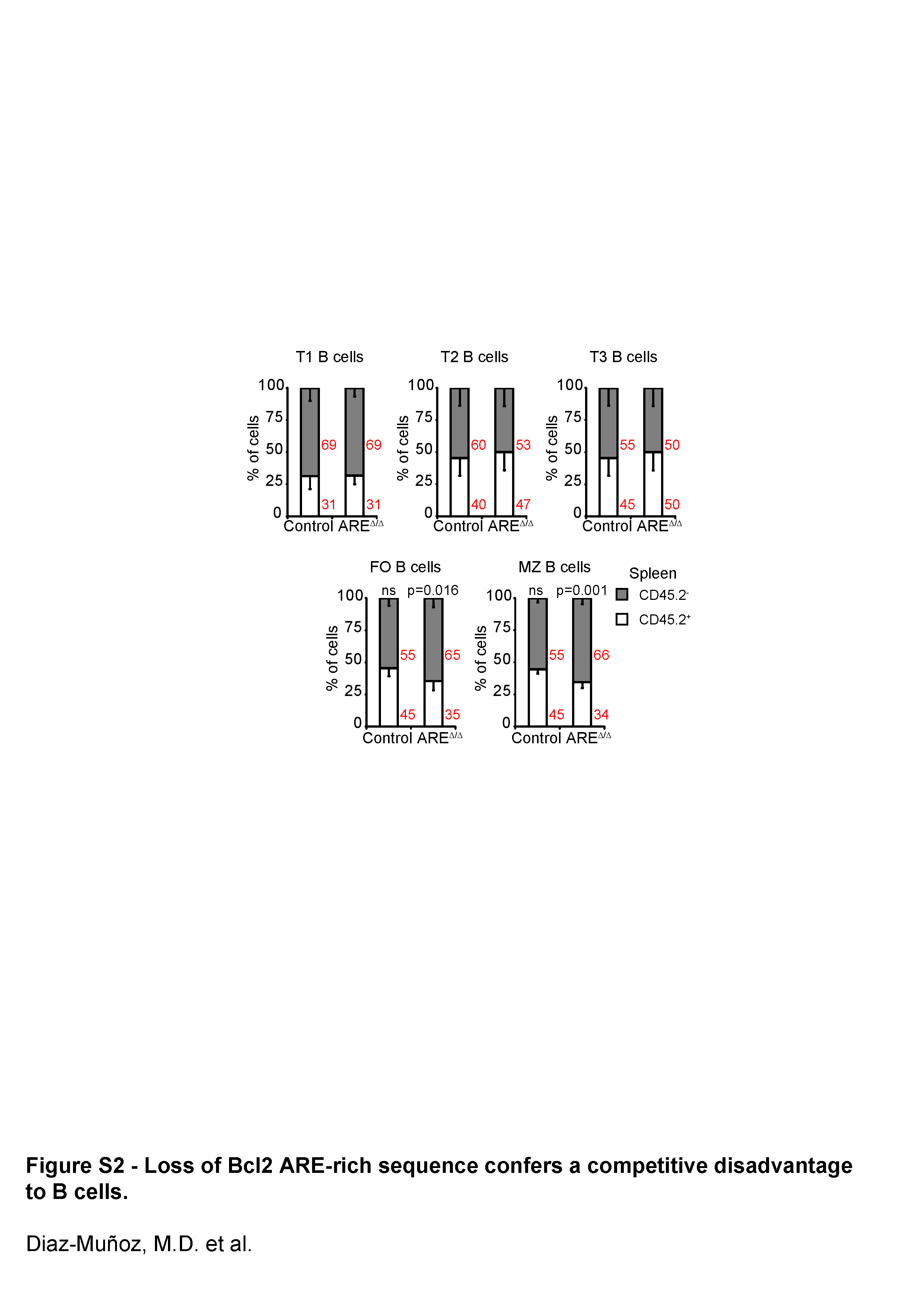

Supplement: S2 Fig — Analysis of the proportions of the different subsets of B cells in the spleen of the competitive bone marrow chimeras described in Fig. 5. Cell populations are defined as: transitional T1 B cells (CD19+ CD93+ IgM+ CD23- cells), transitional T2 B cells (CD19+ CD93+ IgM+ CD23+ cells), transitional T3 B cells (CD19+ CD93+ IgMlow CD23+ cells cells), FO B cells (CD19+ CD93- CD23+ CD21+ cells) and MZ B cells (CD19+ CD93- CD23low CD21high cells). A Mann-Whitney non parametric test was performed for statistical analysis of the data. P values are indicated. n = 8–9 mice per genotype. (TIF) [file pone.0116899.s002.tif]

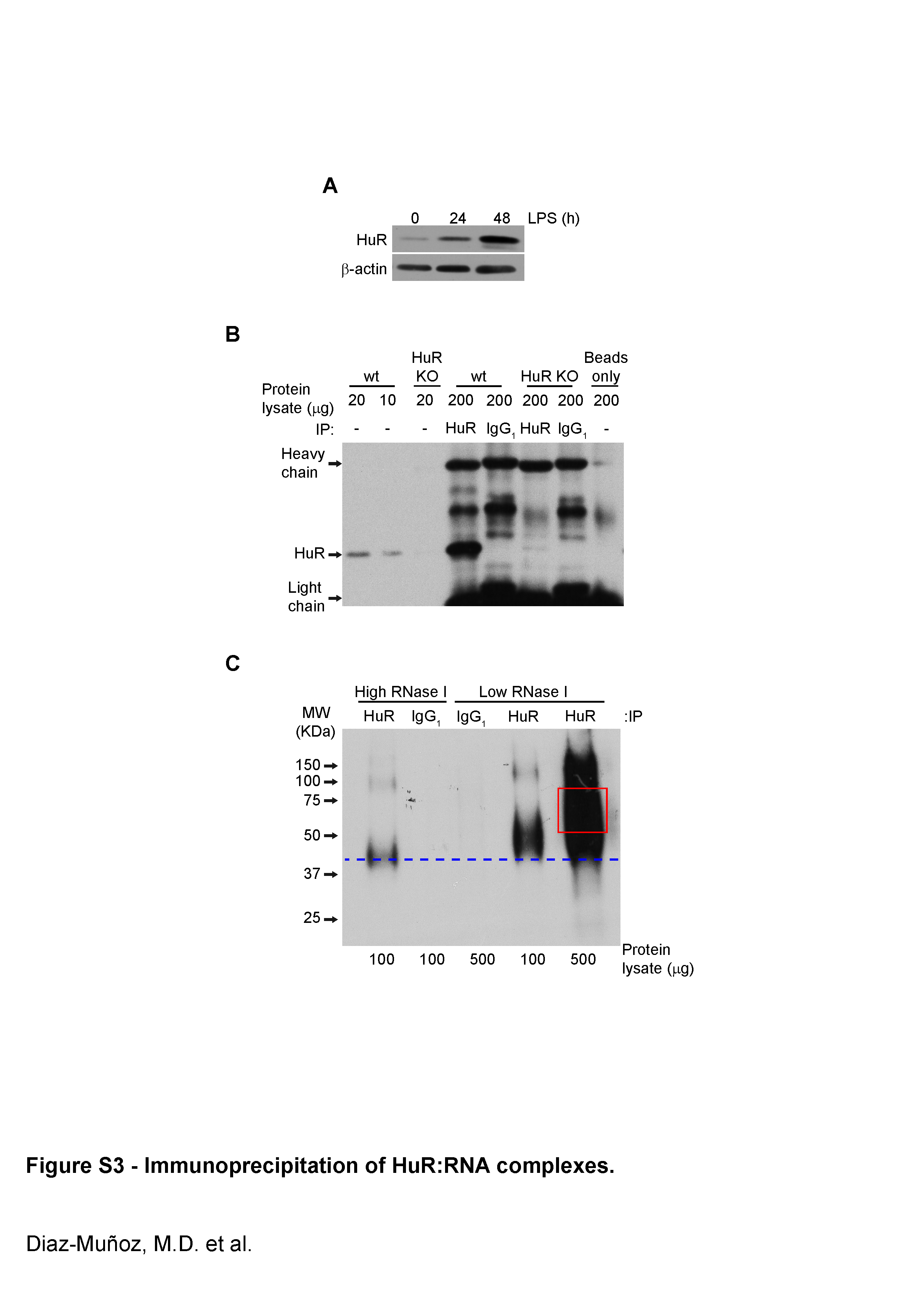

Supplement: S3 Fig — A, Analysis by Western Blot of HuR protein expression in freshly isolated splenic B cells and in B cells activated with LPS for 24 or 48 hours. β-actin is used as loading control B, Validation of HuR immunopreciptation. Splenic B cells from wild-type and HuRflox/flox x mb1cre mice were stimulated with LPS for 48h before isolation of the total protein extracts used in the immunoprecipitation assays. 2 μg of a mouse IgG1 against HuR (3A2 clone, Santa Cruz) or 2 μg of an isotype mouse IgG1 (MOPC21 clone, Sigma Aldrich) were used as indicated in Material and Methods. C, Representative x-ray film detecting radioactive labelled- HuR:RNA complexes. Total cell extracts from LPS-activated B cells irradiated with UV-light (150 mJ/cm2) were used to immunoprecipitate the HuR:RNA complexes after partial RNA digestion with RNase I. The same antibodies described in B were used for the immunoprecipitation and HuR:RNA complexes were detected after RNA labelling with ATP-gamma-32P. The dot line indicates the molecular weight of highly digested RNA molecules cross-linked to HuR. HuR:RNA complexes with approximately a molecular weight from 55 to 80 KDa (red box) were isolated for cDNA library preparation. (TIF) [file pone.0116899.s003.tif]

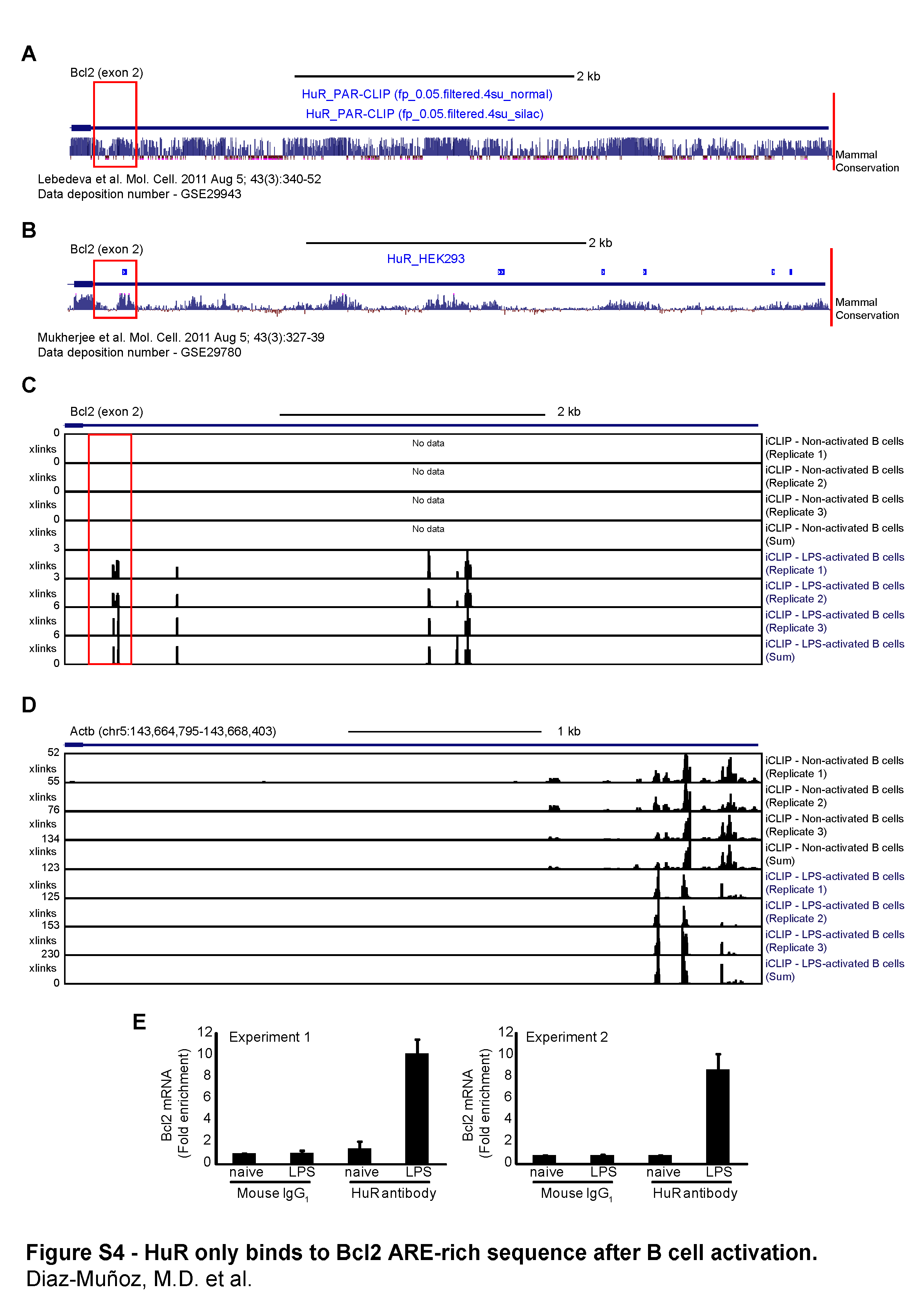

Supplement: S4 Fig — A, B, Analysis of HuR-Bcl2 mRNA interaction in HeLa and HEK293 cells. PAR-CLIP data from Lebedeva et al. (Mol. Cell. 2011 Aug 5;43(3)340–52) and Mukherjee et al. (Mol. Cell. 2011 Aug 5;43(3):327–39) was visualised using the UCSC genome browser and hg18 (A) and hg19 (B) respectively. Bcl2 ARE-rich sequence is indicated by a red box. C, Identification in primary B cells of HuR binding sites across the Bcl2 3’UTR. iCLIP data from three independent iCLIP experiments performed using protein extracts from freshly isolated B cells or LPS-activated B cells were visualised using the UCSC genome browser and mm9 genome annotation. Sum data of the three iCLIP experiments per condition are also shown. D, Mapped iCLIP data along the Actb gene is shown as experimental control. E, Validation of HuR-Bcl2 mRNA interaction by RNA immunoprecipitation assays. Total protein extracts from freshly isolated splenic B cells or cells treated with LPS for 48 hours were used for HuR:RNA immunoprecipitation using 2 μg of a mouse IgG1 against HuR (3A2 clone, Santa Cruz) or 2 μg of an isotype mouse IgG1 (MOPC21 clone, Sigma Aldrich) as negative control. Bcl2 mRNA associated to HuR was detected by qPCR. Data from two independent experiments are shown as mRNA fold enrichment relative to the IgG1 IP controls. (TIF) [file pone.0116899.s004.tif]
