# Supplementary material for: Deletion of AU-Rich Elements within the Bcl2 3′UTR Reduces Protein Expression and B Cell Survival In Vivo
Source: PLoS One. 2015 Feb 13;10(2):e0116899. doi: 10.1371/journal.pone.0116899 (PMC4332480; doi:10.1371/journal.pone.0116899)
Supplement: S1 Methods — (DOCX) [file pone.0116899.s008.docx]

# Extended materials and methods

## Generation of the Bcl2-ARE^flox/flox^ mouse by recombineering.

Briefly, a 12.2 kb sequence containing the exon 2 of Bcl2 was cloned from BAC: RP23-405G16 into the pSC3Z vector. This pSC3Z vector was previously modified to introduce two homology arms to allow recombination in EL350 bacteria. Homology arms 1 and 2 were generated by PCR using the same BAC as DNA template. DNA cloning was performed after enzymatic digestion of the PCR fragments and the empty pSC3Z vector with EcoRI, XhoI and BamHI. The resulted plasmid after homology recombination in EL350 bacteria was called pSC3Z-Bcl2-unmodified. Two additional plasmid vectors were created following a similar strategy, in order to introduce loxP sites flanking the ARE rich sequence of Bcl2. Firstly, the pGEM-loxP-Neo-loxP vector was modified to introduce the homology arms 3 and 4, which flank a neomycin-selection cassette. The restriction enzymes MluI, SpeI (for cloning of the homology arm 3), BglII and SacII (to introduce the homology arm 4) were used. In order to improve mouse genotyping by Southern blot, the 5' primer used to generate the homology arm 3 contained a point mutation to create a new restriction site for BsoBI (absent in the wt allele). The resulting vector was called pGEM-Bcl2 Arms 3&4. Secondly, the plasmid vector pACN-FRT-tACE-Flp-PolII-Neo-FRT-loxP was modified to add the homology arms 5 and 6. The restriction enzymes used for this purpose were KpnI, SalI (to clone the homology arm 5), NheI and SacII (to add the homology arm 6). Homology arms 5 and 6 were cloned flanking the cassette FRT-tACE-Flp-PolII-Neo-FRT-loxP. This cassette allows Flp-mediated recombination in chimeric mice. The resulting plasmid vector was called pACN-Bcl2 Arms 5&6.

In order to introduce a loxP site at the 5’ end of the Bcl2 ARE, the pGEM-Bcl2 Arms 3&4 was digested using MluI and SacII. The linear DNA sequence containing the neomycin-selection cassette flanked by the homology arms 3 and 4 was electroporated (1.75 Kv, 25 µF, 200 Ω) into EL350 bacteria containing the pSC3Z-Bcl2-unmodified plasmid vector. Before electroporation, EL350 bacteria were cultured at 32^o^C in LB media until they reached exponential growth. Expression of exo, bet and gam (the genes involved in homology recombination) was turned on by switching temperature to 42^o^C for 15 minutes. Next, cells were prepared for electroporation by removing salts after several washes with highly-pure water. Selection of positive clones was performed after bacteria culture on LB-Agar plates containing kanamycin (12.5 µg/ml). The modified pSC3Z-Bcl2-loxP-Neo vector was purified and sub-cloned into Bm25.8 bacteria which constitutively express Cre recombinase. Selection of colonies with a loxP-recombined vector (called pSC3Z-Bcl2-loxP) was carried out after enzymatic digestion of purified DNA with NcoI. The pSC3Z-Bcl2-loxP plasmid vector was introduced in EL350 bacteria by electroporation (1.75 Kv, 25 µF, 200 Ω). Selection of positive clones was carried out using ampicillin.

Introduction of the loxP site at the 3’end of the Bcl2 ARE was performed as follows. First, linear DNA containing the FRT-tACE-Flp-PolII-Neo-FRT-loxP cassette flanked by the homology arms 5 and 6 was obtained by enzymatic digestion of the pACN-Bcl2 Arms 5&6 plasmid vector with KpnI and SacII. This linear fragment was electroporated into EL350 bacteria containing the pSC3Z-Bcl2-loxP vector. Bacteria were prepared as indicated above in order to induce homologous recombination. Positive clones were selected after DNA plasmid digestion with NcoI. The final plasmid vector was called pSC3Z-Bcl2-loxP-FRT which contains the final construct for gene targeting of ES cells.

Extraction of the targeting construct from the pSC3Z-Bcl2-loxP-FRT vector was performed after double enzymatic digestion with FspA1 and HpaI. The linear targeting construct was electroporated into Bruce6 ES cells, which were further selected with neomycin. Positive targeted ES cells were identified by Southern blot using DNA probes generated by PCR. 2-4 positive ES cells were microinjected into mouse blastocysts from white-coated C57BL/6-Tyr mice in order to generate chimeric mice. Finally, FRT recombination was performed by breeding chimeric mice with FLPe-tg mice.

Primer sequences to generate all homology arms and DNA probes for Southern blot are described in Table S1. Genomic location of PCR amplicons (GRCm38.p1 annotation) is also stated in the table. BAC: RP23-405G16 was used as DNA template. All restriction enzymes were purchased from NEB or Roche. DNA visualization on agarose gels was performed using ethidium bromide (Sigma). DNA extraction from agarose gels or bacterial extracts was performed using DNA extraction kits from Qiagen.

## Mouse genotyping

Genotyping of targeted ES cells and Bcl2-ARE^flox/flox^ transgenic mouse lines was performed either by Southern blot or PCR. Genomic DNA was extracted from cells or mouse tails following the protocol from the Puregene DNA purification kit (Qiagen). Proteinase K was purchased from Roche. For Southern blot assays, DNA was digested using BsoBI restriction enzyme (overnight at 37°C). Then, DNA was separated in a 0.8% agarose gel and transferred to Hybond XL nylon membrane (GE Healthcare). DNA probes were generated by PCR and labelled with ^32^P-dCTP using the Rediprime II DNA Labelling System (GE Healthcare). The DNA probe was purified using Illustra Nick columns (GE Healthcare). Hybridization and detection by autoradiography was performed as previously described.

Mouse genotyping by qPCR was performed to test germline recombination of the Bcl2-ARE sequence in Bcl2-ARE^flox/flox^ x mb1cre mice. Two different qPCR assays were designed. Assay A amplifies the intron-exon junction of Bcl2, which was not targeted. Assay B amplifies a region of 100 nucleotides inside the ARE, which it is flanked by loxP sites. qPCR assays were performed using 15 ng. of cDNA template and following the instructions from the manufacturer (SYBR Green PCR Master Mix, Life Tech). Relative DNA copy levels were calculated by applying the following formula (X_A_ =2^40-Ct value^ for assay A and X_B_ =^240-Ct value^ for assay B). In order to control for differences in PCR efficiency on assays A and B, a sample from a C57BL/6 mouse or a Bcl2-ARE^flox/flox^ x mb1^wt^ mouse was carried out as positive control in all experiments. Relative DNA copy levels for these positive controls were calculated similarly (C_A_ =2^40-Ct value^ for assay A and C_B_ =2^40-Ct value^ for assay B). The ratio B/A was calculated as follow: B/A ratio = XB * CA / XA * CB. Primer sequences are described in Table S1.
